# High dose interleukin-2 registry, PROCLAIM™; higher center-specific IL-2 dose density correlates with higher response rates

**DOI:** 10.1186/2051-1426-1-S1-P67

**Published:** 2013-11-07

**Authors:** Michael K Wong, Gregory A Daniels, Howard L Kaufman, David F McDermott, Michael A Morse, Sandra Aung, James N Lowder

**Affiliations:** 1 Medicine Division of Hematology-Oncology, University of California, San Diego, CA, USA; 2 Medicine and Solid Tumors USC Norris Comprehensive Cancer Center, University of Southern California, San Diego, CA, USA; 3 Cancer Center, Rush University, Chicago, IL, USA; 4 Biological Therapy and Cutaneous Oncology, Beth Israel Deacones Medical Center, Boston, MA, USA; 5 Medicine, Duke University Medical Center, Durham, NC, USA; 6 Clinical Development and Medical Affairs, Prometheus Labs, San Diego, CA, USA

## Background

High dose interleukin-2 (Proleukin®) is an FDA-approved treatment for metastatic melanoma (mM) and renal cell carcinoma (mRCC) that consistently delivers durable CRs in up to 10% of patients. PROCLAIM™ is a HD IL-2 observational database that was initiated in 2011 to capture current practices, update toxicities and outcomes with current data, and to generate and test new hypotheses. We used retrospective data comprised of 267 patients (mM,170; mRCC, 97) treated between 2007-2012 to explore the hypothesis that IL-2 dose density/week positively correlates with patient outcome. Analysis according to the absolute number of doses/week received by individual patients did not correlate with response, which is congruent with historical data. Unexpectedly, analysis according to the absolute number of doses/week delivered by individual treatment sites did positively correlate with outcome. The data segregated into 6 sites [High Density] that consistently clustered around a median 11 and 9 doses in Cycle 1 and 2, respectively and 3 sites [Low Density] around a median of 8 and 5 doses in Cycle 1 and 2, respectively (p value < 0.0001) . The overall durable response rate (ORR) was 13% for the Low Density cluster vs. 24% for the High Density cluster; the stable disease rate was 13% vs. 21% respectively. No deaths were reported in this retrospective cohort in either cluster.

## Discussion

The seemingly paradoxical finding between IL-2 dose density per patient vs. density per site can be explained by the hypothesis that High Density sites possess the practice pattern and expertise to support IL-2 patients through to the threshold dose density necessary for that individual to trigger a beneficial anti-tumor immune response. These data suggest that the site’s willingness to tolerate elevated creatinine, bilirubin, and thrombocytopenia, and to use vasopressors for hypotension allows for higher number of doses that optimizes outcomes for this group of patients.

## Conclusion

Administering HD IL-2 to maximize the number of safely administered doses/cycle may be important in achieving optimal outcomes. Changes in IL-2 practice should await the analysis of an appropriately sized prospective cohort to test this hypothesis.

